# Supplementary material for: Steric accessibility of the N-terminus improves the titer and quality of recombinant proteins secreted from Komagataella phaffii
Source: Microb Cell Fact. 2022 Sep 5;21:180. doi: 10.1186/s12934-022-01905-2 (PMC9444097; doi:10.1186/s12934-022-01905-2)
Supplement: Supplementary file 1 — Additional file 1: Figure S1. Structural rendering of unmodified RBD and SpyTag-RBD. [file 12934_2022_1905_MOESM1_ESM.zip › Figure S1.pdf]

Steric accessibility of the *N*-terminus improves the titer and quality of recombinant proteins secreted from *Komagataella phaffii*

Figure S1

Neil C. Dalvie[1,2]*, Christopher A. Naranjo[2]*, Sergio A. Rodriguez-Aponte[2,3]*, Ryan S. Johnston[2], J. Christopher Love[1,2]^

[1]Department of Chemical Engineering, Massachusetts Institute of Technology, Cambridge, MA 02139

[2]The Koch Institute for Integrative Cancer Research, Massachusetts Institute of Technology, Cambridge, MA 02139

[3]Department of Biological Engineering, Massachusetts Institute of Technology, Cambridge, MA 02139

*Contributed equally

^Correspondence to clove@mit.edu

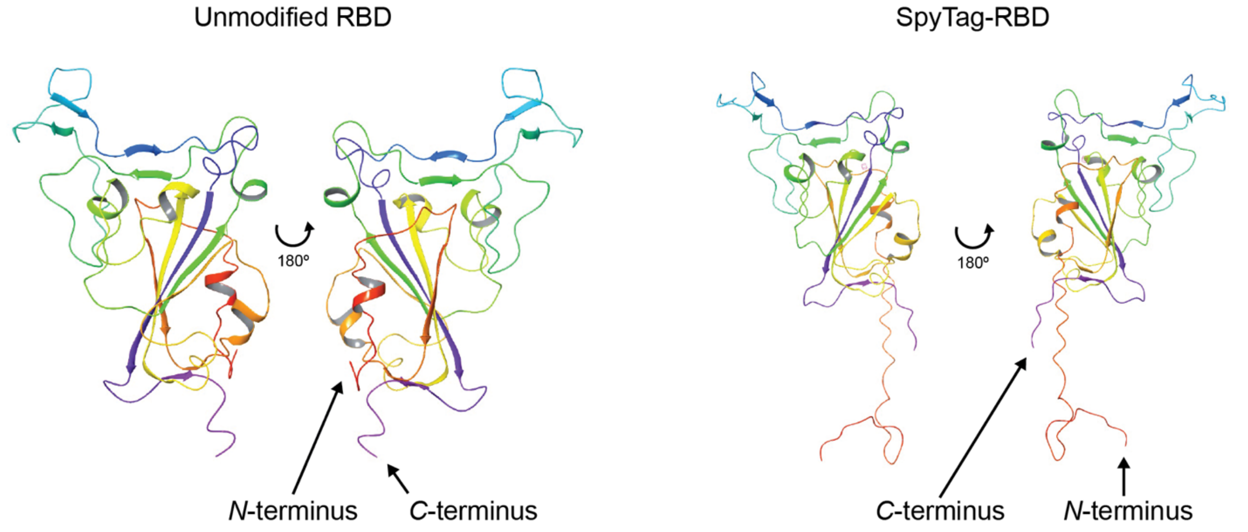

Fig. S1. Structural rendering of unmodified RBD and SpyTag-RBD.
